# Epidemiological Profile and Vascular Risk Factors of Stroke Patients in a Moroccan Provincial Hospital: A Retrospective Study

**DOI:** 10.1155/nri/5135244

**Published:** 2026-02-06

**Authors:** Nadia Mountaj, Anas El Anssari, Mohamed El Assal, Meriem Ouadrhiri, Asmaa Chaib, Yassine Chaib, Mustapha Boucetta

**Affiliations:** ^1^ Department of Nursing Sciences, Higher Institute of Nursing Professions and Health Techniques (ISPITS), Meknes Annex, Fez, Morocco; ^2^ Faculty of Sciences, Biology and Health Laboratory, Ibn Tofail University, Kenitra, Morocco, uit.ac.ma; ^3^ Department of Nursing Education, Higher Institute of Nursing Professions and Health Techniques (ISPITS), Rabat, Morocco; ^4^ Faculty of Sciences, Laboratory of Natural Resources and Sustainable Development, Ibn Tofail University, Kenitra, Morocco, uit.ac.ma; ^5^ Faculty of Medicine and Pharmacy, Biomedical and Translational Research Laboratory, Sidi Mohamed Ben Abdellah University (USMBA), Fez, Morocco; ^6^ Department of Nursing Education, Higher Institute of Nursing Professions and Health Techniques (ISPITS), Kenitra, Morocco; ^7^ Health Sciences and Techniques Team, Higher Institute of Nursing Professions and Health Techniques (ISPITS), Fez, Morocco; ^8^ Faculty of Medicine and Pharmacy of Fez, Laboratory of Clinical Neurosciences, Sidi Mohamed Ben Abdellah University (USMBA), Fez, Morocco

**Keywords:** haemorrhagic stroke, impairment of consciousness, ischaemic stroke, Morocco, prognostic factors, risk factors: hypertension, stroke

## Abstract

Stroke is one of the leading causes of mortality and morbidity worldwide, and its burden is particularly high in low‐ and middle‐income countries. In Morocco, epidemiological data on stroke subtypes, risk factors, symptoms and early outcomes remain limited. The objective of this study was to better understand stroke profiles in Morocco and identify the predictors of haemorrhagic stroke and poor in‐hospital prognosis. A retrospective observational study was conducted including 360 patients admitted to Hospital Mohamed V, Meknes, Morocco. Sociodemographic information, vascular risk factor, clinical presentation, stroke subtype and in‐hospital outcomes were collected. Comparisons were made among patients with ischaemic stroke, haemorrhagic stroke and transient ischaemic attack (TIA). Multivariate logistic regression analyses were performed to identify the independent predictor of haemorrhagic stroke and separately predictors of poor prognosis among ischaemic stroke patients. Ischaemic stroke was the most prevalent subtype (94.2%), followed by haemorrhagic stroke (4.7%) and TIA (1.1%). The mean age was 67.6 ± 13.0 years. The most common vascular risk factors were hypertension (68.6%), diabetes (35.8%) and cardiopathy (51.4%). Diabetes was more frequent among ischaemic stroke patients, while hypertension characterized all haemorrhagic stroke cases. Male sex independently predicted haemorrhagic stroke (OR = 3.27; 95% CI: 1.11–9.65; *p* = 0.032). However, diabetes showed a strong inverse association (OR = 0.082; 95% CI: 0.011–0.636; *p* = 0.017). Overall, in‐hospital prognosis for ischaemic stroke was favourable in 86.7% of cases, and sequalae occurred in 6.2% and mortality in 7.1%. Disturbance of consciousness showed a borderline association with poor prognosis (OR = 2.41; 95% CI: 0.93–6.23; *p* = 0.070). However, age, sex, hypertension, diabetes and cardiopathy were not independent predictors. The findings indicate that most strokes in Morocco are ischaemic and primarily linked to vascular risk factors, particularly hypertension and diabetes. Male sex increases the likelihood of haemorrhagic stroke, while diabetes shifts risk toward ischaemic stroke. Early neurological severity—especially altered consciousness—is the strongest prognostic indicator.

## 1. Introduction

Stroke is one of the leading causes of mortality and morbidity worldwide and is a significant public health concern with substantial social and economic ramifications [1]. The Global Burden of Disease project estimates that globally every year, there are approximately 12 million incident cases and 6.5 million deaths due to stroke with approximately 80%–90% of these cases being ischaemic strokes [2]. The worldwide prevalence of stroke is increasing partly as a result of population aging, increasing urbanization and increasing numbers of modifiable vascular risk factors [3], which is noted in both high‐income countries and low‐/middle‐income countries and particularly in the Middle East and North Africa as well as higher rates in lower resource areas [2, 4].

In LMICs such as Morocco, and specifically in North Africa, rates of hospitalization related to strokes are rapidly increasing, primarily due to the inadequate management of conditions associated with increased stroke risk, such as uncontrolled hypertension/diabetes/cardiovascular diseases [4, 5].

Hypertension remains the primary modifiable risk factor for all forms of stroke (both ischaemic and haemorrhagic) as it leads to endothelial dysfunction, atherosclerosis and small vessel disease [6], while diabetes mellitus predisposes individuals to ischaemic events by increasing the process of atherogenesis and causing microangiopathy [7]. Cardiac pathologies (in particular atrial fibrillation) are responsible for many cases of cardioembolic stroke [8]. Conversely, haemorrhagic strokes are often associated with long‐standing poorly controlled hypertension and are frequently found among male patients, indicating different underlying pathophysiological processes for each subtype of stroke [6, 9].

Apart from risk factors, the early neurological condition of the patient will be a key predictor of short‐term prognosis after a stroke. Many studies have shown that patients with alterations in consciousness and greater neurological deficits immediately following a stroke are more likely to die in hospital or have an early functional decline than those with similar comorbidities (less severe deficits will not necessarily correlate with improved outcomes) [10–12].

While stroke data continue to increase in Morocco, currently there is very limited published information concerning the epidemiological profile, risk factors, clinical presentations and in‐hospital outcomes for stroke patients. Moreover, there are only a small number of studies that have evaluated the association of stroke subtype and in‐hospital prognosis using multivariate analysis.

Acute ischaemic stroke is a condition of clinical and pathophysiological diversity and not a single disease process. The classification of acute ischaemic stroke has numerous causes, for example, cardioembolic stroke, large artery atherosclerosis stroke, small vessel (lacunar) infarction, stroke due to other identified causes and stroke of unknown origin [13, 14]. There are significant variations in the mechanism for each of the above subtypes, the risk factor profile associated with each subtype, the severity of clinical presentation, the management and therapeutics utilized with each subtype, and the short‐ and long‐term outcomes for the subtypes [15].

Research is providing mounting evidence, suggesting that the importance of the distinction of ischaemic stroke subtypes within studies of ischaemic stroke aetiology, to enable the identification of significant relationships between risk factors and outcomes, has not been determined. Specifically, cardioembolic strokes tend to have poorer early prognosis when compared to lacunar infarctions that tend to have less severe manifestations of the disease and better early prognosis. Therefore, the classification of stroke aetiology continues to be acknowledged as a major focus of stroke epidemiological data and outcome research.

In a majority of low‐ and middle‐income nations like Morocco, due to the lack of advancement in diagnosis techniques and incomplete documentation in medical records, routine classification of ischaemic stroke based on the aetiology remains a challenge to the day‐to‐day management of patients. Consequently, many studies of ischaemic stroke done in hospitals have resulted in reporting ischaemic stroke as a single category, while the data generated from these studies should support the interpretation of aetiology, the information provides population evidence of the burden of illness and risk factors associated with stroke.

The aim of this study was to (1) describe the epidemiology and clinical characteristics of stroke patients admitted to a tertiary hospital in Morocco, (2) to compare the distributions of risk factors for different subtypes of stroke and (3) to identify independent predictors of haemorrhagic stroke and poor in‐hospital prognosis.

## 2. Methods

In the present study, we examined a sample of medical records from the Mohammed V Provincial Hospital located in Meknes. This is a secondary‐level service provider that provided services to both urban and rural communities.

This study was authorized by the hospital’s administration. The medical records included individuals aged 18 years and older who had presented at the hospital with the clinical symptoms indicating an acute cerebrovascular event (i.e., stroke) as confirmed by means of a physical examination and/or neuroimaging study performed between January and December of 2024. Patients were divided into three groups based on the type of cerebrovascular event that they were experiencing (1) ischaemic stroke, (2) haemorrhagic stroke and (3) transient ischaemic attack (TIA).

Of the medical records reviewed, patients who did not provide any information regarding specific elements (e.g., age, gender, type of stroke and in‐hospital outcomes) of their medical records were not evaluated and were excluded from the sample. Information was extracted from each patient’s medical record using a standard abstraction form for the following categories of variables: sociodemographic characteristics (e.g., age, sex, occupation, health insurance status and residence), clinical variables (e.g., type of stroke, initial clinical presentation and level of consciousness), vascular risk factors (e.g., hypertension, diabetes, dyslipidaemia, cardiopathy and history of stroke) and in‐hospital outcomes.

Statistical analyses of the data were performed using IBM SPSS Statistics, Version 30 (IBM Corp., Armonk, NY, USA). Descriptive statistics were computed (mean ± SD; range) for continuous variables and count and percent for categorical variables. Statistical comparisons were made between the three groups of strokes: (1) ischaemic stroke, (2) haemorrhagic stroke and (3) TIA. Statistical tests used for categorical variables consisted of the chi‐square test or Fisher’s exact test. Tests used for continuous variables included one‐way ANOVA.

Multivariable logistic regression analysis was employed to determine independent associations between the independent variables. A logistic regression model using as the dependent variable haemorrhagic versus ischaemic stroke, and as independent variables age, sex, hypertension, diabetes, dyslipidaemia and cardiopathy was created. Due to the low number of cases of haemorrhagic stroke and the presence of nearly total separation for certain predictors (e.g., hypertension), the initial full model was found to be unstable. Because the initial full model showed instability due to sparse data patterns, variables with inadequate distribution were removed and a reduced model was constructed.

A second multivariable logistic regression model examining only patients who had ischaemic stroke was created to identify the predictors of poor in‐hospital outcomes, defined as sequelae or mortality versus favourable outcomes (discharge without sequelae). The independent variables included age, sex, hypertension, diabetes, cardiopathy and consciousness disturbance upon admission. Given the sample size and the limited number of events, the conclusions drawn from these analyses should be interpreted with caution. A two‐tailed *p* value < 0.05 was considered statistically significant for all analyses.

The logistic regression models were developed to identify associations rather than to produce a diagnostic or predictive classification tool; therefore, performance metrics such as positive predictive value, negative predictive value and diagnostic accuracy were not calculated.

This research used anonymized data from routine care that were collected by the hospital. The hospital administration approved the project, and there were no personally identifiable data included in the analysis and reporting. The study complied with the ethical standards of the Declaration of Helsinki.

## 3. Results

The study used information from a total of 360 participating patients that ranged in age on average of 67.6 ± 13.0 years (Table 1). The majority of participants were female at 54.2% and made up less than half of the participants at 45.8% (Table 1).

**TABLE 1:** Sociodemographic characteristics and hospital stay duration of patients admitted for acute stroke.

		*n* (%)
Age	*m* ± SD	67.6 ± 13.01

Sex	Female	195 (54.2)
	Male	165 (45.8)

Occupation	Unemployed	33 (9.2)
	Employed	327 (90.8)

Healthcare coverage	AMO	266 (73.9)
	CNSS	94 (26.1)

Residence	Rural	254 (70.6)
	Urban	106 (29.4)

Stay duration	*m* ± SD	8.49 ± 7.72

Total		360 (100.0)

The majority of the patients (90.8%) were in the workforce or had some type of career, while only 9.2% of patients were unemployed. The majority of patients (73.9%) received their medical benefits from AMO, while the remaining patients received their medical benefits from CNSS (26.1%).

Most patients (70.6%) lived in rural areas, while only 29.4% of patients lived in urban areas. The average length of stay in the hospital for all patients was 8.49 ± 7.72 days.

Hypertension was the most prevalent vascular risk factor, affecting 247 (68.6%), while 113 (31.4%) were not hypertensive. Or 129 (35.8%) patients had diabetes, whereas 231 (64.2%) were no diabetic. Hypertension had a significant association with cardiopathy (51.4%). Cardiopathy is the most common complication associated with cardiovascular disease according to the State of the Illness.

Dyslipidaemia had been recorded in 37 (10.3%); the remaining patients had not recorded dyslipidaemia (89.7%). There were 46 (12.8%) patients with a prior stroke, and 87.2% had not experienced a stroke previously (Table 2).

**TABLE 2:** Distribution of vascular risk factors.

		*n* (%)
Hypertension	No	113 (31.4)
	Yes	247 (68.6)

Diabetes	No	231 (64.2)
	Yes	129 (35.8)

Dyslipidaemia	No	323 (89.7)
	Yes	37 (10.3)

Cardiopathy	No	175 (48.6)
	Yes	185 (51.4)

Recurrent stroke	No	314 (87.2)
	Yes	46 (12.8)

Total		360 (100.0)

In the 360‐patient cohort, 339 (94.6%) experienced ischaemic strokes, 17 (4.7%) experienced haemorrhagic strokes and 4 (1.1%) had TIAs. The mean age was similar across stroke subtypes; there were no differences when comparing mean ages (*p* > 0.05).

The distribution of sex among patients with different subtypes of strokes was statistically significant (*p* = 0.050), with a relatively higher percentage of males among those with haemorrhagic strokes (Table 3).

**TABLE 3:** Comparison of demographic and vascular risk factors across subtypes.

		Ischaemic stroke *n* (%)	Haemorrhagic stroke *n* (%)	AIT *n* (%)	Test	*p* value
Age (years)	*m* ± SD	67.65 ± 12.920	66.59 ± 15.273	67.75 ± 13.769	*F* (2, 357) = 0.054	0.948

Sex	Female	189 (96.9)	5 (2.6)	1 (0.5)	Fisher’s exact test	**0.05** ^∗^
	Male	150 (90.9)	12 (7.3)	3 (1.8)		

Hypertension	No	111 (98.2)	0 (0.0)	2 (1.8)	Fisher’s exact test	**0.013** ^∗^
	Yes	228 (92.3)	17 (6.9)	2 (0.8)		

Diabetes	No	212 (91.8)	16 (6.9)	3 (1.3)	Fisher’s exact test	**0.027** ^∗^
	Yes	127 (98.4)	1 (0.8)	1 (0.8)		

Dyslipidaemia	No	302 (93.5)	17 (5.3)	4 (1.2)	Fisher’s exact test	0.279
	Yes	37 (100.0)	0 (0.0)	0 (0.0)		

Cardiopathy	No	3 (1.7)	11 (6.3)	161 (92.0)	Fisher’s exact test	0.218
	Yes	1 (0.5)	6 (3.2)	178 (96.2)		

Recurrent stroke	No	297 (94.6)	14 (4.5)	3 (1.0)	Fisher’s exact test	0.624
	Yes	42 (91.3)	3 (6.5)	1 (2.2)		

Stay duration (days)	*m* ± SD	8.52 ± 7.778	9.18 ± 7.222	3.75 ± 0.957	*F* (2, 357) = 0.823	0.440

Total		339 (94.6)	17 (4.7)	4 (1.1)		

*Note: m*, mean; SD, standard deviation. The significance level (*p* value) of the results obtained after data analysis was set at 0.05 (5%). The bold p values indicate statistically significant differences (*p* < 0.05) among stroke sub.

^∗^ *p* < 0.05 marginally significant, ^∗∗^ *p* < 0.01 significant, ^∗∗∗^ *p* < 0.001 very significant.

Hypertension was significantly associated with the type of stroke experienced (*p* = 0.013); every person with a haemorrhagic stroke had hypertension. Diabetes also differed by the type of stroke (*p* = 0.027). The number of patients with dyslipidaemia and the number of patients having a stroke that recurred were not statistically different (*p* = 0.279 and *p* = 0.624, respectively) (Table 3).

In the instance of ischaemic stroke, cardiopathy was identified in 52.5% of patients; for haemorrhagic stroke, this figure dropped to 35.3%, and for TIAs, the percentage was 25.0%. The difference between ischaemic and haemorrhagic stroke was found not to be statistically significant (Fisher’s exact test, *p* = 0.218).

There were no statistically significant differences in the mean hospital length of stay among the three stroke subtypes (*p* = 0.440) (Table 3).

In the majority of cases (79.4%), hemiplegia (paralysis on one side of the body) was present at initial presentation, whereas hemiparesis (weakness on one side of the body) affected 27.2% of patients. Facial paralysis occurred in 10.3% of patients (Table 4).

**TABLE 4:** Initial clinical manifestations.

Clinical manifestation	*n*	%
Hemiplegia	286	79.4
Hemiparesis	98	27.2
Facial paralysis	37	10.3
Aphasia/speech disorder	53	14.7
Sudden headache	19	5.3
Seizures	9	2.5
Altered consciousness	31	8.6
Sensory loss	1	0.3
Balance disturbance	1	0.3
Visual disturbance	4	1.1

While the above findings were most commonly reported at initial presentation, aphasia (difficulty speaking) occurred in 14.7% of patients. Other nonmotor symptoms included sudden onset headache (5.3%), altered level of consciousness (8.6%), seizures (2.5%) and visual disturbances (1.1%). Sensory loss and problems with balance were uncommon (0.3% each) (Table 4).

All of the above findings point to the fact that motor symptoms were the most prominent feature of the initial clinical picture, which is consistent with how patients typically present with an acute stroke. Table 4 provides further detail about all of the above findings (Table 4).

The vast majority of patients who experienced a stroke did so without residual effects across the different types of ischaemic strokes, with 86.7% achieving a successful outcome, 100% of patients with haemorrhagic strokes and 100% of patients who experienced a TIA achieving a successful outcome. The only cases of residual effects (6.2%) and in‐hospital deaths (7.1%) occurred in the ischaemic stroke group, while there were no recorded neurological sequelae or in‐hospital deaths as a result of haemorrhagic strokes or TIAs (Table 5).

**TABLE 5:** In‐hospital outcome according to stroke subtype.

Discharge outcome	Ischaemic stroke *n* (%)	Haemorrhagic stroke *n* (%)	AIT *n* (%)	Test	*p* value
No sequelae	294 (93.3)	17 (5.4)	4 (1.3)	Fisher’s exact test	0.527
With sequelae	21 (100.0)	0 (0.0)	0 (0.0)		
Deceased	24 (100.0)	0 (0.0)	0 (0.0)		
Total	339 (94.6)	17 (4.7)	4 (1.1)		

While there were apparent differences between groups with respect to the outcomes for discharge, these differences were not statistically significant when compared to the other two stroke categories, as indicated by the Fisher exact test (*p* = 0.527) and limited statistical power due to the low number of haemorrhagic strokes and TIAs (Table 5).

In the multivariate logistic regression analysis, male sex was found to be an independently significant factor for haemorrhagic stroke (OR = 3.27; 95% CI 1.11–9.65; *p* = 0.032). It follows that males had a higher probability of developing a haemorrhagic stroke over an ischaemic one than females. Conversely, diabetes mellitus had an inverse association with the occurrence of a haemorrhagic stroke (OR = 0.082; 95% CI 0.011–0.636; *p* = 0.017), indicating that diabetic individuals are much less likely to develop the haemorrhagic subtype of stroke (Table 6).

**TABLE 6:** Multivariate logistic regression identifying factors associated with haemorrhagic stroke.

	OR (Exp (*B*))	95% CI	*p* value
Male sex	3.27	1.11–9.65	**0.032** ^∗^
Age (years)	0.998	0.962–1.036	0.935
Cardiopathy	0.38	0.13–1.08	0.071
Diabetes	0.082	0.011–0.636	**0.017** ^∗^

*Note:* The bold values highlight variables that were statistically significant in the multivariate logistic regression model (*p* < 0.05), indicating independent associations with haemorrhagic stroke.

^∗^ *p* < 0.05 (marginally significant)

^∗∗^ *p* < 0.01 (significant).

^∗∗∗^ *p* < 0.001 (highly significant).

Cardiopathy and age were not associated with the occurrence of haemorrhagic strokes after the adjustment for other covariates (*p* > 0.05). It is likely that the absence of a significant association for these variables is largely attributable to the low number of haemorrhagic stroke cases available in the study’s sample, which limits the statistical ability to detect and therefore confirm a medium association (Table 6).

None of the traditional vascular risk factors (hypertension, diabetes, cardiopathy) were identified as independent predictors of poor in‐hospital outcomes for patients with the diagnosis of an ischaemic stroke using the multivariate logistic regression model (Table 7).

**TABLE 7:** Multivariate logistic regression identifying predictors of poor in‐hospital outcome in ischaemic stroke.

	OR (Exp (*B*))	95% CI	*p* value
Hypertension	0.68	0.35–1.33	**0.259**
Diabetes	1.13	0.57–2.21	0.730
Cardiopathy	1.70	0.86–3.35	0.126
Male sex	0.76	0.40–1.46	0.413
Disturbance of consciousness	2.41	0.93–6.23	**0.070**
Age (years)	0.997	0.973–1.022	0.813

*Note:* The bold values indicate variables that showed a borderline or statistically significant association with poor in‐hospital outcome in ischaemic stroke in the multivariate logistic regression model (*p* ≤ 0.10), highlighting factors with potential clinical relevance.

The presence of altered level of consciousness during initial presentation appears to have a very weak correlation with negative outcome (OR 2.41; 95% CI 0.93–6.23; *p* = 0.070). The finding suggests that a change in mental status could serve as a clinical marker for impending decline (Table 7).

There was no evidence of age or gender playing a role as prognostic indicators for poor prognosis, once other factors were controlled in the analysis (Table 7).

## 4. Discussion

The aim of this research was to better understand how demographic, medical and outcome‐related factors differ for stroke patients in a cohort of 360 individuals who were admitted to the Hospital Mohamed V, Meknes, Morocco. A total of 94.2% of the patients were diagnosed with ischaemic stroke; 4.7% had haemorrhagic stroke and 1.1% were diagnosed with TIA. These figures support global estimates, indicating that ischaemic stroke represents approximately 80%–90% of all strokes across the world [1, 2]. The mean age of the cohort was 67.6 ± 13.01 years, which is consistent with data from other countries, indicating that stroke incidence is increasing in older individuals [4]. The predominance of women in the cohort is consistent with demographic trends associated with the ageing population.

The most common vascular risk factor in this cohort was hypertension (68.6% of the participants); the second most common vascular risk factor was diabetes mellitus (35.8%); the next most common vascular risk factor was cardiopathy (51.4%); and the least common vascular risk factor was dyslipidaemia (10.3%) [16]. These findings are in line with previous reports from both North Africa and the Middle East, which have found that hypertension remains a significant cause of stroke incidence and mortality in this region [7, 8]. Diabetes mellitus is also commonly found among stroke patients in this region, in accordance with recent research showing that diabetes accelerates the process of atherosclerosis and predisposes patients to ischaemic stroke [6]. The high incidence of cardiopathy suggests that there is an increased awareness of the role played by cardioembolic mechanisms in patients with ischaemic stroke, particularly among older individuals [8, 9].

Among patients with ischaemic stroke in our cohort, cardiac disease was prevalent. Although there were limitations in ascertaining specific cardiac disorders (e.g., atrial fibrillation) due to how cardiopathy was recorded (composite), it is significant that this finding underscores the relevance of cardioembolic mechanisms to this population. Other studies have consistently shown that a cardioembolic stroke (i.e., an ischaemic stroke that has an identifiable cardioembolic source) demonstrates greater neurological deficits and a worse prognosis compared to other subtypes of ischaemic strokes when combined with atrial fibrillation. The reason for the poor prognosis is believed to be the result of larger infarct size, sudden creation of an arterial occlusion and limited collateral circulation. This study supports all the aforementioned findings, although the analyses of each subtype were not possible [17].

Notable example of differences in vascular risk factor profiles among different types of strokes occurred with this cohort. The majority of cases of ischaemic stroke were associated with diabetes mellitus, which is consistent with studies from other countries showing that diabetes mellitus is a leading cause of both atherothrombotic and lacunar infarctions [7, 18]. In contrast, hypertension was found to be more prevalent in cases of haemorrhagic stroke, confirming the critical role that hypertension plays in ruptures of the perforating vessels in the brain, leading to haemorrhagic stroke [10]. The primary clinical features corresponding with the severe acute stroke presentations for all study participants were hemiplegia (49.3%), aphasia (45.7%), facial paralysis (28.6%) and hemiparesis (26.5%), and these three clinical features (hemiplegia, hemiparesis and aphasia) are common in stroke registries or large databases of occurrences [11]. Disturbance of consciousness was not as frequent as the other clinical features and is recognized as a significant marker of neurological severity during the early stages of stroke [10].

### 4.1. Predictors of Haemorrhagic Stroke

Risk factors associated with higher rates of haemorrhagic stroke include male gender (OR = 3.27; *p* = 0.032); this is consistent with reports, which indicate a higher risk of developing intracranial haemorrhage in males due in part to sex‐related differences in vascular biology, sex hormones and exposure to modifiable risk factors such as alcohol consumption [9, 12].

There has been an increased recognition of the role of sex differences between men and women with regard to the occurrence and outcome of stroke. In this analysis, we found that male sex was an independent variable contributing to haemorrhagic stroke risk, while there were no statistically significant differences between sexes with regard to in‐hospital prognosis after controlling for other variables. The current findings support previous studies that found men had a higher risk for developing intracerebral haemorrhages (ICHs), which may be due to the differences in vascular biology or hormonal levels, or increased exposure to various modifiable risk factors associated with stroke risk. Conversely, the impact of female sex on stroke severity and prognosis appears to be more prominent among older age groups, which would help to explain the lack of significant differences between inflated male and female sex prognoses in our current cohort [19].

One risk factor that appeared to have a strong inverse relationship with the risk of developing haemorrhagic and ischaemic stroke was diabetes mellitus (OR = 0.082; *p* = 0.017). Several studies have documented that individuals with diabetes are likely to develop microangiopathy and premature atherosclerosis, thus predisposing them more to ischaemic than haemorrhagic stroke [5]. Age and cardiopathy were not independently associated in our analysis, probably due to the small sample size of the haemorrhagic stroke subgroup (*n* = 17), limiting the ability to detect independent risk factors.

### 4.2. Posthospitalization Outcomes

For all stroke types, most participants were discharged without complications. The only stroke complication (6.2%) and death (7.1%) occurred in the participants who suffered an ischaemic stroke. In the current cohort, there were no poor outcomes for the haemorrhagic stroke group even though they are traditionally linked to worse outcomes. This would appear to be a result of selection bias and possibly having too few mild haemorrhagic cases to demonstrate any significant difference. The lack of significant differences across the stroke subtypes at the time of discharge (*p* = 0.527) emphasizes the limitations associated with small subgroup sizes as well as the impact of initial severity being more important than subtypes alone in predicting discharge outcomes.

There were no patients with haemorrhagic stroke who had any negative outcomes in the current cohort, but this must be interpreted with caution as previous studies have consistently shown that intraventricular extension of ICHs is one of the strongest predictors of poor prognosis. It has been shown that this type of haemorrhage has a higher mortality and creates a patient who has a level of severe neurological impairment. Intraventricular haemorrhage creates acute hydrocephalus, increased intracranial pressure and secondary brain injury, which will significantly detract from the patient’s short‐term outcome. Lack of data about intraventricular extension in this study represents the greatest limitation and likely contributes to the unexpectedly good outcome of patients who had a haemorrhagic stroke [20].

### 4.3. Predictors of Poor In‐Hospital Outcome in Patients With Ischaemic Stroke

All of the unfavourable outcomes were found among patients with ischaemic stroke; therefore, the prognostic analysis was restricted to this subgroup. There were no classical vascular risk factors, including hypertension, diabetes and cardiopathy, that independently predicted a poor in‐hospital outcome; this supports the idea that neurological severity plays a more critical role in the prognosis of acute stroke than chronic comorbidities [12].

Patients with altered consciousness upon admission had a markedly increased likelihood of poor in‐hospital outcome (OR = 2.41; *p* = 0.070). While borderline is statistically significant, this is also of clinical significance, as patients who are admitted with altered consciousness have been found to have larger infarct sizes and more significant brain oedema and risk of early complications, possibly from impaired cerebral perfusion [10].

There was no significant association found between age or sex in terms of in‐hospital prognosis, which may be due in part to having a relatively narrow age range in our cohort of patients with ischaemic strokes.

### 4.4. Comparison With Existing Literature

Our findings support global evidence that supports the predominant role of hypertension and diabetes as drivers of the distribution of stroke subtypes [1, 4, 6]. Moreover, our results support the finding of an inverse association between diabetes and haemorrhagic stroke with respect to prior meta‐analyses showing marked divergence in pathophysiological processes underlying ischaemic and haemorrhagic strokes [11].

The epidemiological profile observed in this Moroccan cohort is consistent with the patterns reported in other hospital‐based stroke registries from low‐ and middle‐income countries, where ischemic stroke remains the leading subtype and hypertension and diabetes persist as the predominant vascular risk factors [5]. There are similar distributions for both stroke classifications and risk factors that have been published in Northern Africa, Middle Eastern countries and sub‐Saharan Africa, which demonstrate that there is an ongoing burden of poorly controllable vascular risk factors in these areas. On the other hand, studies are also reporting variances between the number of haemorrhagic strokes and in clearing the records for in‐hospital results due to the differences between how each study was designed and what access to imaging techniques and level of service of healthcare frequently results [4].

As noted above, the association between neurological alterations due to impaired consciousness has been validated in association with early poor prognosis and increased mortality and dysfunction [10, 11]. Additionally, it has been reported in multiple studies that there is a lack of association between vascular risk factors and early prognosis of patients with stroke, reinforcing the concept that acute severity indicators outweigh the chronic comorbidities in determining early prognosis [12].

For future investigations, translating these results into clinical practice is the goal. Our study highlights the need for future research on a large scale that includes standardized classification of ischaemic stroke subtypes according to their aetiology, as well as standardized evaluation of the severity of ischaemic strokes using validated scales such as the National Institutes of Health Stroke Scale (NIHSS) and the modified Rankin scale (mRS). Further prospective multicentre studies that compare lacunar ischaemic strokes and nonlacunar ischaemic strokes will be beneficial to clarify how the pathophysiologic, clinical presentations and prognosis of lacunar infarctions differ from other ischaemic strokes. In addition, further follow‐up studies to evaluate outcomes and mortality for patients with lacunar infarctions after hospital discharge in a resource‐limited environment are warranted [21].

Table 8 summarizes the main epidemiological characteristics and vascular risk factors identified in the current study and compares them with those reported in recent hospital‐based stroke registries.

**TABLE 8:** Comparison of epidemiological characteristics, vascular risk factors and outcomes of acute stroke in hospital‐based studies.

Author (year)	Country	Study design	Sample size (*n*)	Ischaemic stroke (%)	Haemorrhagic stroke (%)	Main vascular risk factors	In‐hospital outcome
Present study (2024)	Morocco	Retrospective, single‐centre	360	94.2	4.7	Hypertension, diabetes, cardiopathy	Favourable in most cases, poor outcome mainly in ischaemic stroke
Boutayeb et al. (2020) [5]	Morocco	Retrospective, hospital‐based	1020	83.5	16.5	Hypertension, diabetes	High early morbidity; limited outcome data
Sarfo et al. (2018) [4]	Sub‐Saharan Africa	Multicentre registry	3000	68–80	20–32	Hypertension, diabetes	High in hospital mortality
Arboix and Alio (2010) [17]	Spain	Retrospective cohort	2200	78	22	Cardioembolic cardiopathy	Worse prognosis in cardioembolic stroke
Feigin et al. (2021) [2]	Global	GBD analysis	> 12 million	80–85	15–20	Hypertension, diabetes	High global stroke burden
Appelros et al. (2009) [9]	Europe	Systematic review	—	—	—	Sex‐related difference	Higher haemorrhagic risk in men

*Note:* Values are presented as reported in the original publications. Differences across studies may reflect the variations in the study design, population characteristics, diagnostic resources and healthcare systems.

### 4.5. Strengths and Limitations

The study’s strengths are a larger‐than‐average population of patients with ischaemic stroke and use of multivariable logistic regression modelling to identify the strongest predictors. Key limitations of this study include the small number of haemorrhagic stroke cases, the absence of standardised stroke severity scales such as NIHSS or GCS, and the single‐centre design, which may limit the generalisability of the findings.

The study’s retrospective nature did not enable a systematic etiological subtyping of acute ischaemic stroke (a.k.a. cardioembolic, lacunar and atherosclerotic strokes), limiting subtype‐specific evaluations of vascular risk factors and outcome prediction. A cardiovascular (cardiac) disorder variable was created by combining the abovementioned types; thus, it was not consistently possible to validate from the patient’s electronic health record the existence of certain specific cardiac disorders (e.g., atrial fibrillation).

Furthermore, while there was documentation regarding in‐hospital mortality rates, there were inadequate details within the patient medical record to consistently classify a death as being solely due to neurological cause or not due to neurological cause. The multivariable logistic regression analyses were constructed to investigate potential relationships between variables, not to measure diagnostic success, and did not evaluate predictive accuracies (i.e., sensitivity, specificity, etc.); therefore, those measures were not included in the study. Lastly, due to the small number of patients classified as having experienced a haemorrhagic stroke, there was insufficient power for multivariable analyses to provide clinical significance and may have impacted the ability to identify any independent associated variables.

### 4.6. Clinical Implications

It is critical for medical personnel to identify changes in neurological status as early as possible during the hospitalization of patients with a stroke. Our data further highlight the importance of focussing more of the stroke prevention programme on identification of and management of patients with hypertension and diabetes, as well as developing enhanced monitoring of high‐risk patients on admission to facilitate more effective early prognosis stratification and allocation of resources for improved clinical management.

## 5. Conclusion

This research reveals that, within the population studied, the majority of strokes were due to ischaemia, and there exists a significantly higher prevalence of vascular risk factors (primarily hypertension, diabetes and cardiopathy) in stroke patients. The distribution of these major vascular risk factors among the subtypes (ischaemic vs. haemorrhagic) of stroke is in agreement with established pathophysiological mechanisms, wherein the male gender was found to be independently associated with haemorrhagic strokes, while there exists an inverse relationship between diabetes and this subtype of stroke.

The in‐hospital prognosis was found to have a high degree of favourable outcomes; however, all instances of unfavourable prognosis occurred only among patients suffering from ischaemic strokes. The disturbance of consciousness at the time of hospital admission was determined to be the most significant predictor of poor short‐term outcomes via multivariate analysis, indicating that the degree of early neurological severity is more important than traditional vascular risk factors in determining the in‐hospital course of treatment.

In addition to assessing clinical symptoms, imaging studies are also critical indicators of prognosis for stroke patients. In those with ICH strokes, intraventricular bleeding (IVH) is readily recognized as one of the best predictors of poor outcome and death. IVH indicates a larger size of haematoma, higher intracranial pressure and development of secondary Hydrocephalus. Hence, all three of these factors will soon cause early neurological deterioration. While the characteristics of radiology such as IVH were not routinely assessed in the study presented, IVH must be addressed by future studies of prognostic stratification of ICH patients [20].

Therefore, the findings of this study strongly support the need for an increase in the number of healthcare agencies dedicated to providing for the primary prevention of stroke through optimal control of hypertension and diabetes and provide justification for the need to assess and monitor individuals who present to hospitals with altered levels of consciousness in an expedient manner. Furthermore, the findings of this study point to the need for future large‐scale multicentre studies employing standardized neurological severity scales and long‐term follow‐up in order to refine the prognostic models used to guide interventions that are specific to regions.

## Funding

No funding was received for this manuscript.

## Conflicts of Interest

The authors declare no conflicts of interest.

## Data Availability

The data that support the findings of this study are available from the corresponding author upon reasonable request.
